# The factor structure of the Strengths and Difficulties Questionnaire (SDQ) in Greek adolescents

**DOI:** 10.1186/1744-859X-8-20

**Published:** 2009-08-26

**Authors:** George Giannakopoulos, Chara Tzavara, Christine Dimitrakaki, Gerasimos Kolaitis, Vasiliki Rotsika, Yannis Tountas

**Affiliations:** 1 Centre for Health Services Research, Department of Hygiene, Epidemiology and Medical Statistics, Athens University Medical School, Athens, Greece; 2 Department of Child and Adolescent Psychiatry, Athens University Medical School, "Agia Sophia" Children's Hospital, Athens, Greece; 3 Department of Psychiatry, Community Mental Health Center Byron-Kesariani, University of Athens, Athens, Greece

## Abstract

**Background:**

The Strengths and Difficulties Questionnaire (SDQ) is a practical, economic and user-friendly screening instrument of emotional and behavioural problems in children and adolescents. This study was aimed primarily at evaluating the factor structure of the Greek version of the SDQ.

**Methods:**

A representative nationwide sample of 1,194 adolescents (11 to 17 years old) completed the questionnaire. Confirmatory factor analysis (CFA) was conducted to assess the factor structure of the SDQ.

**Results:**

CFA supported the original five-factor structure. The modification of the model provided some improvements. Internal consistency was acceptable for total difficulties, emotional symptoms and prosocial behaviour scale, moderate for hyperactivity/inattention scale and inadequate for peer and conduct problems scale. Older adolescents (aged 15 to 17 years) reported more hyperactivity/inattention and conduct problems than younger ones (aged 11 to 14 years) and girls reported more emotional symptoms and less prosocial behaviour problems than boys. Adolescents of low socioeconomic status (SES) reported more difficulties than those of medium and high SES.

**Conclusion:**

The Greek SDQ could be potentially considered as a community-wide screening instrument for adolescents' emotional and behavioural problems.

## Background

Although the prevalence rates of adolescents' emotional and behavioural problems are high internationally, only a small percentage of adolescents eventually make use of mental health services [1,2]. Validated instruments with the potential to detect children at risk for developing psychosocial problems are, therefore, of crucial importance. Professionals can use such instruments as tools to assess the nature of these problems as a first step for further diagnosis, to prioritise cases as well as to evaluate the effects of an intervention [3-5].

The Strengths and Difficulties Questionnaire (SDQ) is a brief instrument developed primarily for screening purposes, such as selecting at risk cases for further assessment and treatment [6]. The SDQ has been translated into more than 40 languages in recent years, meeting the need for a practical, economic and user-friendly instrument. Versions are available for self-reporting by 11 to 16 year olds as well as for parents and teachers of 4 to 16 year olds. The SDQ, compared to other similar instruments such as the Child Behaviour Checklist (CBCL) [7], seems equally valid for most clinical and research applications [4,8-10]. Additionally, the SDQ is much shorter in format and quick in completion time and as such more convenient for epidemiological studies and screening of large groups of children from the general population. However, the CBCL has been shown to be more appropriate for the investigation of a broader range of psychopathology [9].

The originally proposed factor structure of the SDQ includes five scales of five items each, corresponding with the domains of psychopathology and personal strengths it intends to measure [6]. These scales generate scores for conduct problems, hyperactivity/inattention, emotional symptoms, peer problems, and prosocial behaviour. A total difficulties score is calculated by totalling the four adjustment problems scales (that is, all except for prosocial behaviour). An extended version of the SDQ includes an impact supplement that enquires further about chronicity of the problems, distress, social impairment and burden for others.

A number of mainly European studies have provided consistent support for its proposed five-factor structure [11], although numerous secondary loadings mainly on the prosocial behaviour factor or limited associations between the item 'I usually do as I am told' and the theoretically associated conduct problems factor have been reported [5,12-14]. Other studies have failed to replicate the originally hypothesised five-factor structure [15,16]. However, few studies have used confirmatory analytic techniques to evaluate the original hypothesised factor structure of the SDQ [17-21].

Similar to the findings concerning the SDQ factor structure, there is a substantial body of rather varied research with regard to other psychometric properties of the instrument [5,10,22-26].

In Greece, few published studies have investigated emotional and behavioural problems among school-aged children and adolescents [27] due to the relative lack of screening instruments that have been developed or adapted in the Greek language against agreed scientific attributes [28-32]. As the European interest in mental health prevention and health promotion services is expanding, availability of good quality instruments at a national level becomes ever more necessary. The present study is the first to examine the factor structure of the SDQ through confirmatory analytic techniques.

## Methods

### Participants and procedures

This study was conducted in the year 2003 within the framework of the European project 'Screening and Promotion for Health-Related Quality of Life (HRQoL) in Children and Adolescents - A European Public Health Perspective' [33]. The school sampling in Greece was random, multistaged and performed to take into account distribution of the target population by age and administrative school region. The target population was adolescents aged 11 to 17. A sample size of 1,800 adolescents was considered necessary to detect a minimally important difference of half a standard deviation (SD) in HRQoL scores within each age strata between children with and without special healthcare needs or a chronic condition. A response rate of approximately 70% was expected, so the initial sample size was set at 2,400 children and adolescents. In Greece, ages 11 to 17 correspond to six secondary school grades. Approximately 400 students were included from each of the 6 age groups/grades in order to reach the original target of 2,400 adolescents. For example, the total number of students in Greece attending the first grade of the secondary school is 119.055. If an administrative region had a total number of 2,174 students attending the first grade of the secondary school then 8 students were randomly recruited from a school in that region ((2,174 × 400)/119,055 = 7.6 students). Each age group/grade had been calculating accordingly, for each sector. Schools in each sector were randomly selected by a computer program and students of each selected school were selected randomly from classroom name lists. A sample of 1,900 adolescents (11 to 17 year olds) was recruited. A total of 1,194 (that is, a 63% response rate) of self-reported questionnaires (479 boys and 715 girls) were returned. Total adolescent sample mean age was 13.6 years (SD = 1.7). Regarding the socioeconomic status (SES) characteristics of the sample, 37.59% came from low-income families, 44.96% came from middle-income families and 17.45% from high-income families. Students were asked to complete the questionnaire at home after providing written informed consent. Inclusion criteria were adequate reading skills. Previous research on the representativeness of the present sample has reported that non-responder interviews showed no significant differences between responders and non-responders with regard to adolescents' and parents' general perceived health, parents' marital status and highest educational level, and type of residence, indicating that a selection bias is less likely [33].

### Measures

The SDQ contains 25 items (small sentences), categorised into 5 scales of 5 items each: hyperactivity/inattention, emotional symptoms, conduct problems, peer problems and prosocial behaviour [6]. Responses to each of the 25 items consisted of 3 options: not true, somewhat true, or certainly true. For all scales the items that are worded negatively are assigned scores of 2 for certainly true, 1 for somewhat true, and 0 for not true. The version for youths was used in the present study. In order to combat inherent weaknesses of crosscultural adaptation (for example, semantic and scale equivalence) the research team in the present study followed a standardised translation methodology according to international crosscultural translation guidelines [34]. To assess familial SES, the Family Affluence Scale (FAS) [35] was used, addressing issues of family car ownership, having their own unshared room, the number of computers at home and times the children spent on holiday in the past 12 months. The FAS was collected in seven categories (from 0 the lowest to 7 the highest) and was re-coded into three groups for the analysis (low FAS level (0 to 3), intermediate (4 to 5) and high (6 to 7)). The psychometric properties of the FAS are acceptable and support its use as a self-reported adolescents' measure [36].

### Statistical analysis

Confirmatory factor analysis (CFA) with maximum likelihood procedure was used to assess the theoretical model for the SDQ proposed by Goodman [13]. For all models, independence of error terms was specified, and the factors were allowed to be correlated. A number of approaches were used to assess the fit of the CFA models, including the comparative fit index (CFI), the goodness of fit index (GFI), the χ^2 ^goodness of fit test and the root mean square error of approximation (RMSEA) [37]. There are a variety of guidelines for interpreting the fit of a specific model based on these indices. For the CFI and GFI indices, values close to or greater than 0.95 are taken to reflect a good fit to the data [38]. RMSEA values of less than 0.05 indicate a good fit, and values as high as 0.08 indicate a reasonable fit [38]. CFA was carried out using the SPSS AMOS package (SPSS, Chicago, IL, USA).

Internal consistency reliability was determined by calculation of the Cronbach α coefficient. A minimum reliability of 0.70 for measures used in-group comparisons has been recommended [39]. Multivariate analysis of variance (MANOVA) using the Tukey correction for multiple comparisons was used to explore differences on SDQ scales according to gender and age groups, as well as differences on SDQ scales according to SES.

## Results

### CFA

Fit indices resulting from the CFA for the five-factor hypothesised model were estimated. The CFI, GFI and RMSEA values were 0.78, 0.91 and 0.057, respectively, indicating a questionable fit. Factor loadings were evenly distributed from 0.19 to 0.66. A further analysis was performed according to modification index (MI), which was used to suggest potential improvements to the model. The MI is employed to measure how much the χ^2 ^test is reduced when a particular change in the model is suggested. A large sample size can make even a small MI value significant. Therefore, only the largest MI is analysed in this text. Estimates of the modified model are presented in Table 1. The item 'I usually do as I am told' loaded moderately (with a loading of 0.19) on the conduct problems scale and had secondary loadings of -0.25 on emotional symptoms scale. The item 'I think before I do things' had loading equal to 0.25 on hyperactivity/inattention scale and a secondary loading was added on prosocial behaviour scale (with a loading of -0.23). The modification indices also suggested an additional error covariance between the items 'I am easily distracted; I find it difficult to concentrate' and 'I finish the work I am doing; my attention is good'. An additional error covariance was also observed between the items 'I am restless; I cannot stay still for long' and 'I am constantly fidgeting or squirming'. These findings indicated that the aforementioned items create a subdimension within the hyperactivity/inattention factor. After the effects of modification, CFA revealed an acceptable fit according to the following results for CFI, GFI and RMSEA: 0.92, 0.95, and 0.04, respectively.

**Table 1:** Parameter estimates from the results of confirmatory factor analyses of the Strengths and Difficulties Questionnaire (SDQ) five-dimensional model

	Prosocial	Hyperactivity	Emotional	Conduct problems	Peer problems
I try to be nice to other people. I care about their feelings.	0.58				
I usually share with others (food, games, pens and so on).	0.43				
I am helpful if someone is hurt, upset or feeling ill.	0.64				
I am kind to younger children.	0.48				
I often volunteer to help others (parents, teachers, and/or children).	0.66				
I am restless; I cannot stay still for long.		0.46			
I am constantly fidgeting or squirming.		0.55			
I am easily distracted; I find it difficult to concentrate.		0.51			
I think before I do things.	-0.23	0.25			
I finish the work I am doing. My attention is good.		0.44			
I get a lot of headaches, stomach aches or sickness.			0.41		
I worry a lot.			0.59		
I am often unhappy, downhearted or tearful.			0.65		
I am nervous in new situations. I easily lose confidence.			0.46		
I have many fears, I am easily scared.			0.51		
I get very angry and often lose my temper.				0.64	
I usually do as I am told.			-0.25	0.19	
I fight a lot. I can make other people do what I want.				0.44	
I am often accused of lying or cheating.				0.58	
I take things that are not mine from home, school or elsewhere.				0.49	
I am usually on my own. I generally play alone or keep to myself.					0.53
I have one good friend or more.					0.42
Other people my age generally like me.					0.43
Other children or young people pick on me or bully me.					0.46
I get on better with adults					0.41

### Internal consistency reliability

The internal consistency coefficient for the total difficulties score was 0.77. Cronbach α values for the prosocial behaviour, emotional symptoms and hyperactivity/inattention were 0.72, 0.73 and 0.63, respectively. The lowest α was found on the peer problems scale (0.50) and conduct problem scale (0.56).

### Effects of age, gender and SES

According to the MANOVA results (Table 2), girls reported higher mean scores on the emotional symptoms and prosocial behaviour scales. Age differences revealed that those aged 15 to 17 years reported higher mean scores on the conduct problem and hyperactivity/inattention scales compared to those aged 11 to 14 years. The score on the prosocial behaviour scale was lower in children aged 15 to 17 years than those aged 11 to 14 years. A gender/age interaction was found for the emotional symptoms scale. Females had higher scores on the emotional symptoms scale than males and this difference was greater in the age group of 15 to 17 years than in the age group of 11 to 14 years.

**Table 2:** Means and standard deviations (SD) of the Strengths and Difficulties Questionnaire (SDQ) scales

		11 to 14 years		15 to 17 years	
		
	Total	Male	Female	Male	Female
Prosocial behaviour^a^	8.1 ± 1.8	7.9 ± 2.0	8.4 ± 1.6	7.5 ± 2.1	8.2 ± 1.8
Hyperactivity^b^	3.6 ± 2.2	3.1 ± 2.2	3.1 ± 2.1	3.9 ± 2.2	4.1 ± 2.2
Emotional symptoms^abc^	3.0 ± 2.1	2.3 ± 2.2	3.1 ± 2.0	2.5 ± 2.0	3.7 ± 2.1
Conduct problems^b^	2.0 ± 1.5	3.0 ± 1.7	2.8 ± 1.4	3.0 ± 1.5	3.1 ± 1.5
Peer problems	1.8 ± 1.7	1.9 ± 2.0	1.7 ± 1.6	1.9 ± 1.8	2.0 ± 1.6

^a^*P <*0.05 for gender differences; ^b^*P <*0.05 for age differences; ^c^significant gender/age interaction.

MANOVA showed significant differences in SDQ scores between adolescents of low versus high SES and low versus medium SES (Table 3), except for the conduct problems and prosocial behaviour scores. No significant differences were obtained between adolescents of high versus medium SES.

**Table 3:** Differences in Strengths and Difficulties Questionnaire (SDQ) scales according to family affluence level

	Family affluence scale (FAS)					
	
	Low (0 to 3)		Medium (4 to 5)		High (6 to 7)	
	
	Mean T value	SD	Mean T value	SD	Mean T value	SD
Emotional symptoms^ab^	51.82	10.04	49.28	9.76	48.60	9.94
Conduct problems	50.49	10.02	49.55	10.28	50.39	9.35
Hyperactivity^ab^	51.20	9.35	50.08	9.78	49.21	10.43
Peer problems^ab^	51.69	10.60	48.91	9.57	48.77	9.14
Prosocial behaviour	50.61	9.40	49.36	10.39	49.63	10.77

^a^*P *< 0.05 for differences between low and medium; ^b^*P *< 0.05 for differences between low and high.

SD = standard deviation.

## Discussion

The main objective of the present study was to investigate the originally proposed five-factor structure of the self-report Greek SDQ. The analysis confirmed the proposed five-factor structure of the instrument [13], although some modifications seemed to be necessary in order to gain an acceptable model fit.

The present report is one of the few studies that has used confirmatory analytical techniques to evaluate the original hypothesised factor structure of the SDQ. Dickey and Blumberg [17] could not entirely confirm the predicted five-component structure of the parent-report SDQ and extracted a stable three-factor model consisting of externalising problems, internalising problems, and a positive behaviour factor. A Dutch study of parent and teacher reports [18] showed that the model with five latent variables outlined by Goodman fitted only moderately well, whereas the model with three latent variables (that is, externalising behaviour, internalising behaviour, and prosocial behaviour) did not show better fit indices. Three studies have reported confirmatory factor analyses of the self-report SDQ. A study in a general population of Russian adolescents [19] concluded that the proposed five-factor solution had inadequate psychometric properties with low factor loadings and scale reliabilities (0.44 to 0.70). Similarly, an Irish study in a community-based education sample reported many deviations from the hypothesised component structure of the self-report SDQ, particularly in relation to the reverse-coded items [20]. Unlike the above-mentioned findings, D'Acremont and Van der Linden [21] suggested a reliable factor organisation of the French version of the teacher-report SDQ with acceptable to very good scale reliabilities (0.64 to 0.90).

In the present study, consistent with previous research [5,12,14], the item 'I usually do as I am told' showed limited association with the originally associated conduct problems scale. In fact, being obedient seemed to be indicative of more emotional symptoms in the present population, suggesting possible cultural differences in the interpretation of this item content. Moreover, the item 'I think before I do things' was found to load moderately on the originally associated hyperactivity/inattention scale and load more strongly on the prosocial behaviour scale. This finding has been reported elsewhere [5,12,14,20] and lends further support to previously reported deviations from the originally proposed structure in relation to the reverse-coded items (that is, positively worded items of adjustment problems scale). It could be suggested that a more valid scoring procedure in the Greek population may exclude the aforementioned items (that is, 'I usually do as I am told' and 'I think before I do things') from the scoring system. Furthermore, the hyperactivity/inattention scale appeared to be divided into two subdimensions (that is, hyperactivity and inattention). This finding may reflect the inability to assess the multidimensional attention deficit hyperactivity disorder through only five items different in nature to each other [20].

With regard to the internal consistency of the instrument, Cronbach α values for the total difficulties, emotional symptoms, and prosocial behaviour scales were found to be above the minimum recommended level of 0.70 indicating acceptable internal consistency. However, internal consistency for the hyperactivity/inattention scale was moderate and a low α was revealed for peer and conduct problems scales, agreeing well with other research [11,13,16,19,20,22].

Previous studies on the SDQ [10,22-24] have consistently shown that adolescent girls report more emotional symptoms and less prosocial behaviour problems than boys, while boys have a higher score on conduct and peer problems scales. Parents seem to report less prosocial behaviour problems for their adolescent daughters and more hyperactivity/inattention for their adolescent sons [10,24,25]. Moreover, analyses on the age effects on the SDQ scales scores provide somewhat mixed findings depending mainly on cultural differences. However, studies do suggest that during adolescence, hyperactivity/inattention and peer problems significantly decrease with age, while emotional symptoms increase, especially in girls [5,22,23,26]. Finally, it has been reported that adolescents with a less favourable social and economic background show significantly more total difficulties and score significantly higher on the hyperactivity/inattention and peer problems scales of the SDQ [26]. However, some studies have not shown a marked effect of family economic disadvantage on adolescents' emotions or behaviour [25].

The present analysis revealed that girls reported higher prosocial behaviour and more emotional symptoms (increasing with age); this finding is consistently supported by previous research [10,23,24]. However, unlike results from other studies [5,22,26], it is interesting that in the present sample older adolescents (attending mainly senior high school) reported more hyperactivity/inattention and conduct problems than those aged 11 to 14 (attending mainly junior high school). The particular educational circumstances in Greece may be involved in the observed differences and the effect of the extremely competitive and stressful school environment in Greek senior high schools, due to the intensive 3-year preparation for the entry university exams, should be further explored in future research. By contrast, the current results were in the expected direction in terms of family SES, with adolescents coming from a lower socioeconomic background reporting more emotional and behavioural problems [25,26].

## Conclusion

The present study lends further support to Goodman's five-factor structure via confirmatory factor analytic techniques, suggesting that the original component scales may be appropriate for a sample of Greek adolescents. A scoring procedure that better reflects some modifications in the factor structure of the instrument may improve the ability of school practitioners and clinicians to screen for emotional and behavioural problems among Greek adolescents. It is worth mentioning, however, that the results of this analysis may not be well generalised to clinical samples, as the current study employed a school-based population. The utility of the self-report SDQ in Greek clinical samples of adolescents and the discriminant validity remain to be examined.

## Competing interests

The authors declare that they have no competing interests.

## Authors' contributions

GG, CT, CD, GK and VR participated in the preparation of the paper. YT had overall supervision of the study.
